# Facial cleft? The first case of manitoba‐oculo‐tricho‐anal syndrome with novel mutations in China: a case report

**DOI:** 10.1186/s12887-021-02506-5

**Published:** 2021-01-21

**Authors:** Shuchen Gu, Yimin Khoong, Xin Huang, Tao Zan

**Affiliations:** Department of Plastic and Reconstructive Surgery, Shanghai Ninth People’s Hospital, Shanghai JiaoTong University School of Medicine, 639 Zhizaoju Road, 200011 Shanghai, China

**Keywords:** MOTA syndrome, FREM1 gene, Mutation, Case report

## Abstract

**Background:**

Manitoba-oculo-tricho-anal (MOTA) syndrome is a rare syndrome with only 27 cases reported worldwide so far, but none was reported in the population of Eastern Asia. Such extremely low prevalence might be contributed by misdiagnosis due to its similarities in ocular manifestations with facial cleft. In our study, we discovered the first case of MOTA syndrome in the population of China, with 2 novel FRAS1 related extracellular matrix 1 (FREM1) gene stop-gain mutations confirmed by whole exome sequencing.

**Case presentation:**

A 12-year-old Chinese girl presented with facial cleft-like deformities including aberrant hairline, blepharon-coloboma and broad bifid nose since birth. Whole exome sequencing resulted in the identification of 2 novel stop-gain mutations in the FREM1 gene. Diagnosis of MOTA syndrome was then established.

**Conclusions:**

We discovered the first sporadic case of MOTA syndrome according to clinical manifestations and genetic etiology in the Chinese population. We have identified 2 novel stop-gain mutations in FREM1 gene which further expands the spectrum of mutational seen in the MOTA syndrome. Further research should be conducted for better understanding of its mechanism, establishment of an accurate diagnosis, and eventually the exploitation of a more effective and comprehensive therapeutic intervention for MOTA syndrome.

## Background

Manitoba oculo-tricho-anal syndrome (MOTA syndrome, OMIM 248,450) is a rare syndrome characterized by aberrant hairline, blepharon-coloboma, broad bifid nose with or without anorectal stenosis. It was first reported in 6 children from 4 related families in the Manitoba Indian pedigree by Marles et al. in 1992 [1]. Several published pedigree reports have revealed that the inheritance pattern of MOTA syndrome was primarily autosomal recessive [1, 2]. The pathogenic gene, however, remained ambiguous until 2011, when Slavotinek et al. confirmed that MOTA syndrome was caused by mutations in the gene called FRAS1 related extracellular matrix 1 (FREM1, OMIM 608,944), demonstrated in both patients and mutant mice models [3].

FREM1 gene, located on the chromosome 9p22.3, encodes the basement membrane protein which can potentially affect the craniofacial and renal development [4]. Mutations of this gene may cause 3 distinct clinical phenotypes including bifid nose with or without anorectal and renal anomalies (BNAR syndrome, OMIM 608,980), MOTA syndrome, or trigonocephaly (OMIM 614,485) [3, 5, 6].

Despite its clear genetic etiology and clinical manifestations, currently only 27 cases of MOTA syndrome have been reported worldwide. There has been no single case of MOTA syndrome reported in the Chinese population, likely due to underdiagnosis or misdiagnosis especially in the plastic and reconstructive clinics, since both MOTA syndrome and facial cleft share several similarities in ocular manifestations [7]. In this study, we reported an analysis of whole exome sequencing from 1 Chinese girl with a highly concordant clinical phenotype of MOTA syndrome. The diagnosis of MOTA syndrome was confirmed by the identification of 2 novel stop-gain mutations in the FREM1 gene.

## Case presentation

**Fig. 1:**
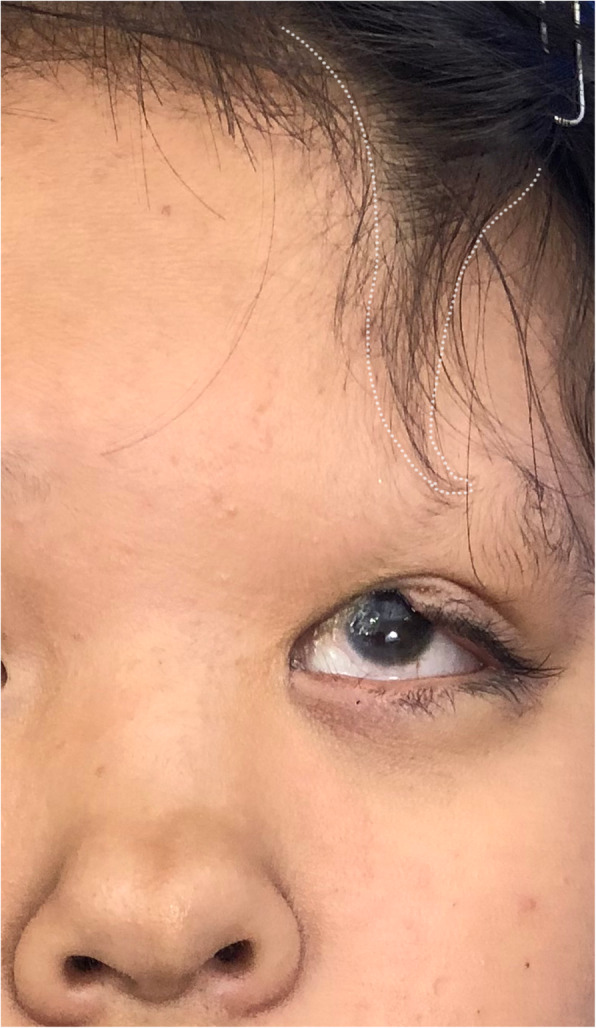
The frontal view of the patient with an aberrant unilateral wedge-shaped anterior hairline and the loss of ipsilateral eyebrow, hypertelorism, ipsilateral medial eyelid colobomas and a broad bifid nose

**Fig. 2:**
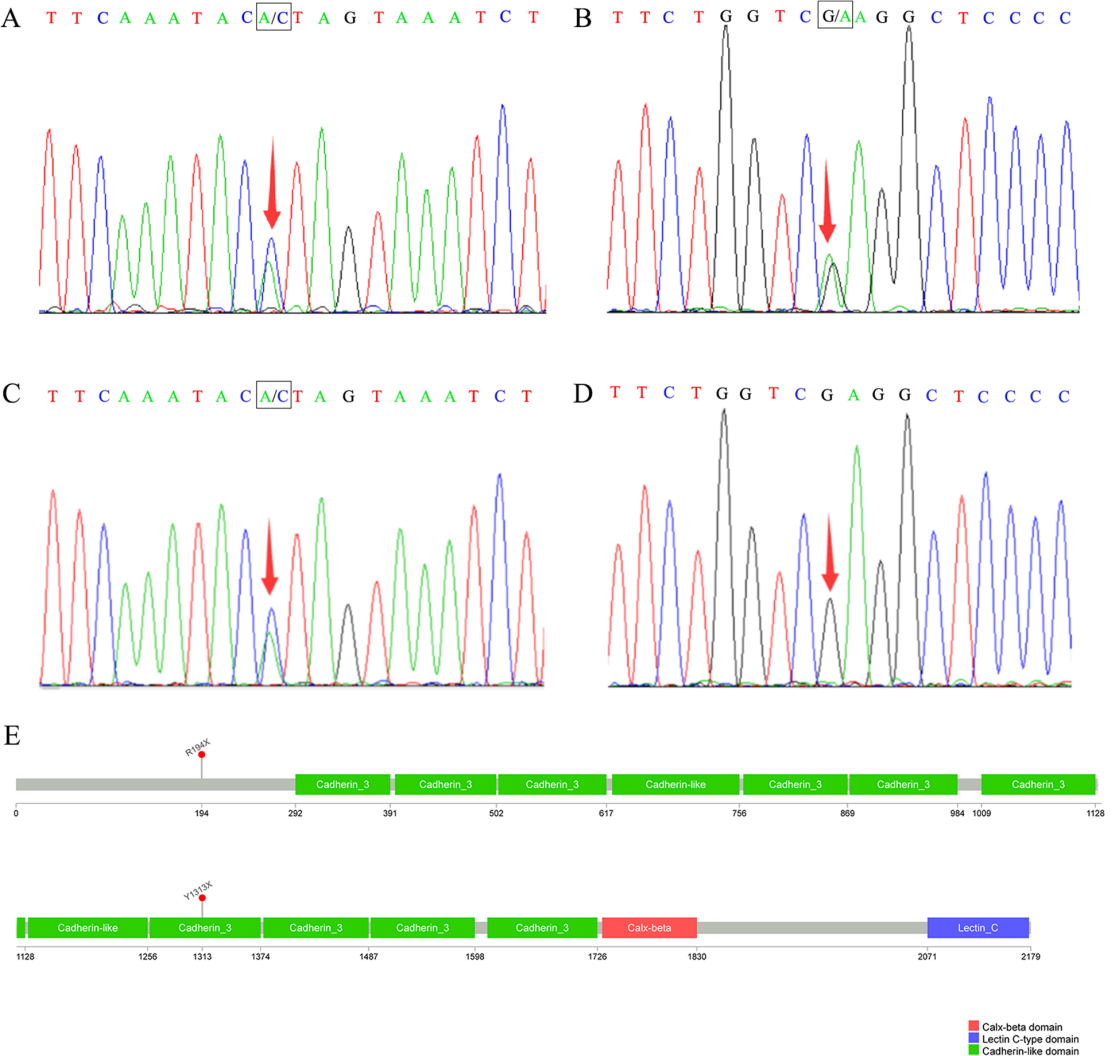
Mutation analysis of the FREM1variant performed on the DNA extracted from the patient (**a, b**) and her father (**c, d**). **a** heterozygous c.3939 A > C (p.Y1313X) variant at exon 23in the patient; **b** heterozygous c.580G > A (p.R194X) variant at exon5 in the patient; **c** heterozygous c.3939 A > C variant at exon 23 in the patient’s father; **d** no variant at exon 5 in the patient’s father; **e** a schematic diagram of FREM1 primary structure

A 12-year-old Chinese girl presented with facial cleft-like deformities existing from birth without any remarkable family history. Physical examination showed an aberrant unilateral wedge-shaped anterior hairline with the loss of ipsilateral eyebrow, hypertelorism, ipsilateral medial eyelid colobomas and a broad bifid nose (Fig. 1). The appearance of her left eyeball was almost normal except for slight symblepharon; the vision was however reduced to light perception. The contralateral eye was completely normal in both appearance and vision. Gastrointestinal anomalies were not found in this patient. Whole exome and Sanger sequencing were performed on blood-extracted DNA from the patient and her father. Two novel stop-gain mutations in the FREM1 gene were found in the patient: heterozygous c.3939 A > C (p.Y1313X) variant at exon 23 (Fig. 2a) and heterozygous c.580G > A (p.R194X) variant at exon5 (Fig. 2b). The former mutation was also found in the healthy father (Fig. 2c), whereas the latter 1 was not (Fig. 2d). Such genetics sequencing results together with the clinical manifestations allowed the establishment of the final diagnosis of MOTA syndrome.

## Discussion and conclusions

Facial cleft is one of the most frequently encountered congenital deformities in the plastic and reconstructive clinics. According to the classification proposed by Paul Tessier in 1976, Tessier number 10 cleft was characterized by coloboma of the middle third of the upper eyelid and divided eyebrow with the vertical part extending to the anterior hairline, which share similar ocular manifestations with the above case [8]. Yet the characteristic of the bifid nose could not be explained based on the diagnosis of Tessier number 10 cleft. Hence, whole exome sequencing was performed and the result revealed 2 novel mutations. According to the criteria above, the diagnosis of MOTA syndrome was therefore confirmed and established. Both of these 2 variants were stop-gain mutations resulted in premature stop codon which could eventually lead to the impairment of the translation of functional proteins. Interestingly, the father shared the same heterozygous c.3939 A > C variant at exon 23 with the patient, which however did not cause any clinical manifestations of MOTA syndrome. This finding indicated that this mutation alone may not be sufficient to cause changes in phenotype. The disease phenotype might be caused by the c.580G > A variant at exon 5.

The fact that both MOTA syndrome and facial cleft sharing similarities in ocular manifestation might have contributed to the chance of misdiagnosis of MOTA syndrome especially by the plastic surgeons. On one side, MOTA syndrome exhibits heterogeneous symptoms and among which there were several phenotypes resembling the features of Tessier number 10 cleft. Chacon-Camacho OF et al. have conducted a systematic review of the 27 reported cases of MOTA syndrome worldwide till 2017 [7], apart from our current report, there has no other new case been reported since then. Of the 28 reported cases of MOTA syndrome to date, 15 merely suffered from facial deformities, and 5 with no observable nasal defect [7]. On the other side, the most frequently seen organ abnormalities in MOTA syndrome, the anteriorly placed anus and anal stenosis could be asymptomatic, thus might easily be neglected by surgeons.

It is crucial to conduct genetic testing to make a definite diagnosis which might also remind plastic surgeons of the potential organ abnormalities. A group of extracellular matrix proteins encoded by FRAS1, FREM1 and FREM2 gene have been shown to form a ternary complex located at the basement membrane [9]. Mutations of FRAS1 and FREM2 could lead to a severe malformation disorder called Fraser syndrome, while FREM1 mutations could result in less severe phenotypes such as the MOTA syndrome and BNAR syndrome. Although this particular case of MOTA syndrome in our report showed no abnormalities in her internal organs, previous reports have revealed that these syndromes could have overlapping clinical symptoms, therefore those mild phenotypes could be coexist with more severe organ abnormalities as well [7].

## Data Availability

The datasets analyzed during the current study are under submission to NCBI ClinVar.

## Abbreviations

MOTA: Manitoba-oculo-tricho-anal
FREM1: FRAS1 related extracellular matrix 1
